# Supplementary material for: Evidence for a unitary structure of spatial cognition beyond general intelligence
Source: NPJ Sci Learn. 2020 Jul 2;5:9. doi: 10.1038/s41539-020-0067-8 (PMC7331750; doi:10.1038/s41539-020-0067-8)
Supplement: Supplementary file 1 — Supplementary Material [file 41539_2020_67_MOESM1_ESM.pdf]

Evidence for a unitary structure of spatial cognition beyond general intelligence

Margherita Malanchini, Kaili Rimfeld, Nicholas G. Shakeshaft, Andrew McMillan, Kerry L. Schofield, Maja Rodic, Valerio Rossi, Yulia Kovas, Philip S. Dale, Elliot M. Tucker-Drob, & Robert Plomin

List of Tables

Supplementary Table 1. Descriptive statistics for each spatial test and *g*, randomly selecting one twin out of each pair
Supplementary Table 2. Sex differences in the six tests part of the spatial orientation battery
Supplementary Table 3. Univariate quantitative sex limitation models for individual tests of navigation included in the Spatial Spy battery
Supplementary Table 4. Confirmatory factor analysis and model fit indices across the six tests of spatial orientation
Supplementary Table 5. Common pathway model across the six tests of spatial orientation
Supplementary Table 6. Comparative model fit indices for the phenotypic models including all 16 tests of spatial skills (6 tests of spatial orientation and 10 tests of object-based spatial skills)
Supplementary Table 7. Hierarchical common pathway model examining the genetic and environmental factors characterizing variation in across the 16 tests of spatial abilities (6 tests of spatial orientation and 10 tests of object-based spatial skills).
Supplementary Table 8. Associations between the time lag of administration between the two batteries and spatial ability measures.

List of Figures

Supplementary Figure 1. Two-factor model of spatial ability separating across the two spatial batteries administered at two different time points and following two different formats (online traditional psychometric assessment vs. virtual environment).
Supplementary Figure 2. Two-factor model of spatial ability separating objects-based and orienting tests combining putatively separate categories of tests administered across the two batteries.
Supplementary Figure 3. Bifactor model. This model allows for the variance common to all tests to load onto a single factor of spatial ability, while separating the variance specific to each of the two batteries
Supplementary Figure 4. Hierarchical model of spatial ability including two first-order factors (object manipulation and navigation/visualization) loading onto a second order general spatial ability factor
Supplementary Figure 5. Cholesky decomposition exploring the genetic and environmental overlap between *g* and the hierarchical model of spatial ability factor
Supplementary Figure 6. One-factor model of spatial ability including the 16 spatial tests accounting for *g* at the level of the indicator (each test).
Supplementary Figure 7. Two-factor model of spatial ability separating the two batteries accounting for *g* at the level of the indicators (each test).
Supplementary Figure 8. Two-factor model of spatial ability separating objects-based and orienting tests combining putatively different aspects of spatial skills across the two batteries accounting for *g* at the level of the indicators (each test).
Supplementary Figure 9. Three-factor model of spatial ability separating objects-based, navigation and visualization tests across the two batteries accounting for *g*.
Supplementary Figure 10. Hierarchical model including three first-order spatial factors (Navigation, Object-based and Visualization) and a second-order common factor of Spatial ability, accounting for *g* at the level of the indicators (each test).
Supplementary Figure 11. Hierarchical model including three first-order spatial factors (Navigation, Object-based and Visualization) and a second-order common factor of Spatial ability, accounting for *g* at the level of the first-order factors.
Supplementary Figure 12. Genetic and environmental variance in a measure of *g* at age 16 and in the common spatial ability factor.

Supplementary Figure 13. Correlations between all spatial tests on the other half of the sample
Supplementary Figure 14. Factor structure of navigation abilities conducted examining the other half of the sample
Supplementary Figure 15. Hierarchical model of spatial abilities conducted in the other half of the sample

Supplementary Tables

Supplementary Table 1. (a) Descriptive statistics for each spatial test and developmental *g*, randomly selecting one twin out of each pair. (b) Descriptive statistics for the other half of the sample.

(a)

	N	mean	sd	min	max	range	skew	kurtosis
Navigation directions (cardinal points	1330	0.000	1.000	-3.220	2.440	5.660	-0.170	-0.300
Navigation landmarks	1278	0.000	1.000	-4.260	1.980	6.240	-1.140	1.610
Map reading	1244	0.000	1.000	-4.180	1.620	5.800	-1.020	1.040
Route memory	1217	0.000	1.000	-4.260	1.510	5.770	-1.350	1.920
Large-scale perspective taking	1285	0.000	1.000	-3.350	1.470	4.830	-0.960	0.400
Large-scale scanning	1229	0.000	1.000	-4.160	1.590	5.750	-1.430	2.120
KC cross-section	927	0.000	1.000	-2.200	2.310	4.510	-0.180	-0.760
KC 2d drawing	920	0.000	1.000	-3.480	1.540	5.020	-0.710	-0.020
KC pattern assembly	896	0.000	1.000	-2.260	2.620	4.870	-0.370	-0.520
KC Elithorne maze	804	0.000	1.000	-3.210	1.990	5.200	-1.160	1.290
KC mechanical reasoning	914	0.000	1.000	-3.060	2.940	6.000	-0.180	-0.050
KC paper folding	888	0.000	1.000	-2.290	2.090	4.390	-0.120	-1.000
KC 3d drawing	833	0.000	1.000	-2.030	2.340	4.370	0.030	-1.000
KC shapes rotation	850	0.000	1.000	-2.330	1.870	4.200	-0.340	-0.850
KC small-scale perspective taking	859	0.000	1.000	-1.640	2.980	4.620	0.610	-0.190
KC mazes	850	0.000	1.000	-3.070	2.650	5.720	-0.140	-0.120
General cognitive ability (g)	1234	0.000	1.000	-2.830	3.040	5.870	0.320	0.010

Note: all measures were standardized and residualized for age and sex within the randomly selected half of the sample by means of linear regression; KC: King's Challenge battery

(b) Descriptive statistics for the other half of the sample

	N	mean	sd	min	max	range	skew	kurtosis
Navigation directions (cardinal points)	1349	0.000	1.000	-3.250	2.790	6.050	-0.110	-0.330
Navigation landmarks	1312	0.000	1.000	-4.330	2.070	6.400	-0.900	0.870
Map reading	1268	0.000	1.000	-4.300	1.620	5.930	-1.000	1.160
Route memory	1234	0.000	1.000	-4.500	1.550	6.050	-1.250	1.710
Large-scale perspective taking	1316	0.000	1.000	-3.450	1.450	4.900	-0.850	0.170
Large-scale scanning	1260	0.000	1.000	-3.960	1.610	5.570	-1.250	1.510
KC cross-section	939	0.000	1.000	-2.250	2.330	4.580	-0.260	-0.710
KC 2d drawing	932	0.000	1.000	-3.230	1.610	4.850	-0.790	0.180
KC pattern assembly	911	0.000	1.000	-2.420	2.130	4.550	-0.440	-0.580
KC Elithorne maze	815	0.000	1.000	-3.850	1.940	5.790	-1.060	1.280
KC mechanical reasoning	921	0.000	1.000	-3.080	2.590	5.680	-0.100	-0.230
KC paper folding	893	0.000	1.000	-2.380	1.900	4.280	-0.270	-0.960
KC 3d drawing	868	0.000	1.000	-2.130	2.240	4.380	-0.090	-1.010
KC shapes rotation	857	0.000	1.000	-2.450	1.920	4.370	-0.420	-0.760
KC small-scale perspective taking	880	0.000	1.000	-1.680	3.180	4.850	0.590	-0.450
KC mazes	862	0.000	1.000	-3.170	2.550	5.720	-0.220	-0.180
General cognitive ability (g)	1242	0.000	1.000	-2.770	2.950	5.720	0.150	-0.150

Note: all measures were standardized and residualized for age and sex within the randomly selected half of the sample by means of linear regression; KC: King's Challenge battery

Supplementary Table 2. Sex differences in the six tests part of the spatial orientation battery

	mean F	Mean M	*F* (df)	*t (p)*	*adj* R_2
Navigation directions (cardinal points)	-0.33 (0.88)	0.50 (0.96)	261.0 (1, 1328)	16.16 (<2e-16)	0.163
Navigation landmarks	-0.16 (0.83)	0.43 (0.69)	178.0 (1, 1276)	13.34 (< 2e-16)	0.121
Map reading	-0.25 (0.90)	0.50 (0.67)	256.9 (1, 1242)	16.02 (<2e-16)	0.170
Route memory	-0.17 (0.89)	0.42 (0.63)	162.6 (1, 1215)	12.75 (< 2e-16)	0.117
Large-scale scanning	0.00 (0.79)	0.25 (0.65)	36.27 (1, 1227)	6.023 (2.2e-09)	0.027
Large-scale perspective taking	-0.13 (0.97)	0.25 (0.89)	49.76 (1, 1283)	7.054 (2.8e-12)	0.036

Note: F = females; M = males; adj = adjusted

Supplementary Table 3a Sex limitation model fitting sub-model comparisons (significant differences are marked in bold). HetACE= quantitative heterogeneity model; HomACE= homogeneity model (no sex differences at all); ep=estimated parameters; minus2LL= minus 2 log-likelihood; df= degrees of freedom; AIC= Akaike information criterion; diffLL= change in log-likelihood; diffdf= change in degrees of freedom (significant differences are marked in bold). (b) Univariate model fitting results for males and females separately

**Total Navigation ability**							
	ep	-2LL	df	AIC	diffLL	diffdf	p
Model: HetACE	8	5863.64	2122	1619.64	-	-	-
Model: HomACE	5	5874.35	2125	1624.35	10.71	3	**0.01**
**Scanning**							
	ep	-2LL	df	AIC	diffLL	diffdf	p
Model: HetACE	8	6992.87	2481	2030.87	-	-	-
Model: HomACE	5	7048.2	2484	2080.2	55.33	3	**< .001**
**Perspective taking**							
	ep	-2LL	df	AIC	diffLL	diffdf	p
Model: HetACE	8	7314.77	2593	2128.77	-	-	-
Model: HomACE	5	7328.79	2596	2136.79	14.02	3	**< .001**
**Navigation landmarks**							
Quantitative differences:							
	ep	-2LL	df	AIC	diffLL	diffdf	p
Model: HetACE	8	7216.09	2582	2052.09	-	-	-
Model: HomACE	5	7248.5	2585	2078.5	32.42	3	**< .001**
**Navigation directions**							

Quantitative differences:

	ep	-2LL	df	AIC	diffLL	diffdf	p
Model: HetACE	8	7392.27	2671	2050.27	-	-	-
Model: HomACE	5	7398.39	2674	2050.39	6.12	3	0.11

Map reading

	ep	-2LL	df	AIC	diffLL	diffdf	p
Model: HetACE	8	6983.5	2504	1975.5	-	-	-
Model: HomACE	5	7079.69	2507	2065.69	96.19	3	**< .001**

Route memory

	ep	-2LL	df	AIC	diffLL	diffdf	p
Model: HetACE	8	6769.48	2443	1883.48	-	-	-
Model: HomACE	5	6894.1	2446	2002.1	124.63	3	**< .001**

Supplementary Table 3b Heritability (A), shared environmental (C) and nonshared environmental (E) estimates for each test calculated separately for females and males. Numbers in parentheses are 95% confidence intervals

	**A**	**C**	**E**
**Females**			
Navigation ability	0.53 (0.29-0.61)	0 (0.01-0.19)	0.47 (0.38-0.56)
Scanning	0.14 (0.01-0.26)	0 (0.01-0.14)	0.86 (0.74-0.99)
Perspective taking	0.33 (0.01-0.43)	0.01 (0.01-0.25)	0.66 (0.57-0.78)
Navigation landmarks	0.35 (0.04-0.53)	0.09 (0.01-0.33)	0.56 (0.46-0.67)
Navigation directions	0.46 (0.21-0.62)	0.08 (0.01-0.28)	0.46 (0.37-0.54)
Map reading	0.27 (0.01-0.37)	0 (0.01-0.23)	0.73 (0.62-0.85)
Route memory	0.26 (0.01-0.36)	0 (0.01-0.25)	0.74 (0.64-0.86)
**Males**			
Navigation ability	0.59 (0.39-0.69)	0 (0.01-0.15)	0.40 (0.31-0.52)
Scanning	0.01 (0.01-0.30)	0.13 (0.01-0.25)	0.86 (0.71-0.99)
Perspective taking	0.22 (0.01-0.36)	0 (0.01-0.10)	0.78 (0.63-0.99)
Navigation landmarks	0.09 (0.01-0.45)	0.25 (0.35-0.40)	0.66 (0.51-0.78)
Navigation directions	0.63 (0.42-0.71)	0 (0.01-0.17)	0.37 (0.29-0.46)
Map reading	0.63 (0.47-0.73)	0 (0.01-0.08)	0.37 (0.26-0.51)
Route memory	0.53 (0.28-0.63)	0 (0.01-0.16)	0.47 (0.37-0.60)

Supplementary Table 4. Confirmatory factor analysis and model fit indices across the six tests of spatial orientation.

**Spatial orientation battery**	**Factor loadings**	**S.E.**
Navigation directions (cardinal points)	0.736	0.017
Navigation landmarks	0.756	0.017
Map reading	0.682	0.019
Route memory	0.636	0.021
Large-scale perspective taking	0.582	0.022
Large-scale scanning	0.566	0.024
Model fit indices: χ_2 = 69.051(9), p< .00005, CFI = 0.972 TLI = 0.953, RMSEA = 0.071 SRMR. =0.026		

Note: CFI = comparative fit index; TLI = Tucker-Lewis index; RMSEA = root mean square error of approximation, SRMR = standardized root mean square residual

Supplementary Table 5. Common pathway model examining the common and specific genetic (A), shared-environmental (C) and nonshared environmental variance (E) across the six tests of spatial orientation and model fit indices.

**Variance specific to each test**				**Percentages of A, C and E variance in each test captured by the common Navigation factor**		
**Measure**	**A**	**C**	**E**	**A**	**C**	**E**
Navigation directions	0.181 (0.136; 0.233)	0.001 (-0.000; 0.001)	0.274 (0.228; 0.324)	66%	100%	36%
Navigation landmarks	0.000 (-0.001, 0.001)	0.038 (0.006; 0.096)	0.394 (0.340; 0.451)	88%	53%	28%
Map reading	0.035 (0.000; 0.130)	0.000 (-0.000; 0.000)	0.506 (0.431; 0.586)	88%	100%	20%
Route memory	0.058 (0.005; 0.167)	0.001 (-0.078; 0.099)	0.564 (0.478; 0.654)	80%	100%	16%
Large-scale scanning	0.000 (-0.001; 0.002)	-0.000 (-0.000; 0.000)	0.705 (0.664; 0.748)	100%	100%	10%
Large-scale perspective-taking	0.047 (0.003; 0.139)	0.000 (0.000; 0.000)	0.597(0.524; 0.675)	82%	100%	15%
**Variance common across all tests**						
Navigation latent factor	0.634 (0.410; 0.912)	0.083 (-0.005; 0.430)	0.278 (0.206; 0.362)			
Model fit indices: AIC = 39177.976; χ_2 = 269.937 (148), p = 0.0000; CFI = 0.968; TLI = 0.971; RMSEA = 0.030 ; SRMR = 0.049						

Note. All paths are standardized and squared, numbers in parentheses are 95% confidence intervals around the estimates; CFI = comparative fit index; TLI = Tucker-Lewis index; RMSEA = root mean square error of approximation, SRMR = standardized root mean square residual

Supplementary Table 6a. Comparative model fit indices for the phenotypic models including all 16 tests of spatial skills (6 tests of spatial orientation and 10 tests of object-based spatial skills)

	**Model**	**CFI**	**TLI**	**RMSEA**	**Chi^2**	**AIC**	**Akaike weights**	**SRMR**	**Correlations between latent factors**
a	1 Factor	0.890	0.873	0.061	692.730 (104), p < .001	41594.511	0.00	0.059	-
b	2 Factors (Spatial Spy battery and. King's Challenge battery)	0.958	0.951	0.037	316.000 (103), p < .001	41232.958	-	0.040	.741
c	2 Factors (Object Manipulation and Spatial Orientation)	0.920	0.907	0.053	529.390 (103), p< .001	41433.108	0.00	0.054	.847
d	Bifactor Model (Spatial Spy battery, King's Challenge battery and spatial ability)	0.822	0.770	0.083	5471.714(120), p< .001	41970.898	0.00	0.196	-
e	3 Factors (Object Manipulation Navigation and Visualization)	0.953	0.944	0.041	351.870 (101), p < .001	41259.589	0.50*	0.041	Obj with Or = .726<br>Or with Sc = .948<br>Sc with Obj = .858
f	3 Factors (Object Manipulation Navigation and Visualization) and a second order common Spatial Ability factor	0.953	0.944	0.041	351.870 (101), p < .001	41259.589	0.50*	0.041	-
g	2 Factors (Object Manipulation and Navigation/Visualization) and a second order common Spatial Ability factor	0.729	0.697	0.095	1554.839(107), p< .001	42450.558	0.00	0.297	-

Note: Akaike weights were calculated using the 'Weights' function of the R package MuMIn (Bartoń, 2019); Model b was not included in the calculation of the Akaike weights as the interpretation of the model is problematic since the two separate factors that emerge may be a product of differences in the two test batteries and the modality of assessment rather than reflecting real clustering of separate overarching abilities. * = these two models are equivalent. Model c specifies a bifactor model; however, in this case this model could also be described as a multi-trait-multi-method (MTMM) approach[1] because, as shown in Figure S3, the two separate test-based factors could reflect specific assessment method variance. CFI = comparative fit index; TLI = Tucker-Lewis index; RMSEA = root mean square error of approximation, SRMR = standardized root mean square residual; AIC = Akaike Information criterion.

Supplementary Table 6b. Comparative model fit indices for the phenotypic models including all 16 tests of spatial skills (6 tests of spatial orientation and 10 tests of object-based spatial skills) including general cognitive ability (*g*) as a correlate to the model.

	Model	CFI	TLI	RMSEA	Chi^2	AIC	Akaike Weights	SRMR	Correlations between latent factors
h	1 Factor *accounting for g*[1]	0.894	0.862	0.067	609.795 (104), p < .001	33880.185	0.00	0.053	-
i	2 Factors (Spatial Spy battery and. King's Challenge battery) *accounting for g*[1]	0.961	0.949	0.041	288.468 (103), p < .001	33560.857	-	0.038	.659
j	2 Factors (Object Manipulation and Spatial Orientation) *accounting for g*[1]	0.925	0.901	0.057	461.763 (103), p < .001	33734.153	0.00	0.051	.775
k	3 Factors (Object Manipulation Navigation and Visualization) *accounting for g*[1]	0.957	0.942	0.043	306.307 (101), p < .001	33582.697	0.50*	0.038	Obj with Or = .633<br>Or with Sc = .949<br>Sc with Obj = .806
l	3 Factors (Object Manipulation Navigation and Visualization) and a second order common Spatial Ability factor *accounting for g*[1]	0.957	0.942	0.043	306.307 (101), p < .001	33582.697	0.50*	0.038	-
m	3 Factors (Object Manipulation Navigation and Visualization) and a second order common Spatial Ability factor *accounting for g*[2]	0.951	0.941	0.044	348.789 (114), p < .001	33599.178	0.00	0.041	-

Note: Akaike weights were calculated using the 'Weights' function of the R package MuMIn (Bartoń, 2019). Model g was not included in the calculation of the Akaike weights as the interpretation of the model is problematic since the two separate factors that emerge may be a product of differences in the two test batteries and the modality of assessment rather than reflecting real clustering of separate overarching abilities. * = these two models are equivalent. [1] = general cognitive ability (*g*) included in the model at the level of the indicators; [2] = *g* included in the model at the level of the first order latent factors. Models i and j and models i and are k represent two different ways f specifying the same model, therefore their model fit indices are identical. CFI = comparative fit index; TLI = Tucker-Lewis index; RMSEA = root mean square error of approximation, SRMR = standardized root mean square residual; AIC = Akaike Information criterion.

Supplementary Table 7. Genetic and environmental variance components for the hierarchical model of spatial abilities

Variance specific to each test			Loading on first order factor	A and E variance captured by the first order factors but not shared with the general spatial ability factor	
**Measure**	**A**	**E**		**A**	**E**
Navigation from directions (cardinal points)	0.136 (0.094; 0.186)	0.255 (0.214; 0.309)	0.777 (0.755; 0.799)	0.030	0.048
Navigation from landmarks	0.072 (0.025; 0.145)	0.419 (0.356; 0.487)	0.712 (0.685; 0.740)	0.025	0.041
Map reading	0.016; (-0.016; 0.133)	0.511 (0.438; 0.588)	0.687 (0.658; 0.717)	0.023	0.036
Route memory	0.059 (0.007; 0.160)	0.565 (0.483; 0.654)	0.612 (0.577; 0.647)	0.018	0.029
Cross-sections	0.075 (0.024; 0.155)	0.556 (0.491; 0.625)	0.606 (0.573; 0.639)	0.100	0.029
2D drawing	0.010 (-0.046; 0.173)	0.470 (0.398; 0.549)	0.721 (0.694; 0.748)	0.139	0.041
Pattern assembly	0.028 (-0.000; 0.128)	0.527 (0.456; 0.602)	0.666 (0.637; 0.696)	0.100	0.029
Shapes rotation	0.051 (0.009; 0.125)	0.476 (0.412; 0.543)	0.688 (0.658; 0.718)	0.128	0.036
Mechanical reasoning	0.150 (0.093; 0.221)	0.473 (0.405; 0.546)	0.614; (0.579; 0.648)	0.100	0.029
Paper folding	0.107 (0.059; 0.169)	0.366 (0.302; 0.429)	0.726 (0.698; 0.754)	0.143	0.042
3D drawing	0.093 (0.046; 0.155)	0.303 (0.250; 0.362)	0.777 (0.752; 0.801)	0.160	0.047
Small-scale perspective taking	0.154 (0.081; 0.250)	0.602 (0.512; 0.697)	0.494 (0.453; 0.535)	0.000	0.000
Large-scale scanning	0.000 (-0.001; 0.002)	0.741 (0.698; 0.783)	0.509 (0.468; 0.550)	0.000	0.000
Large-scale perspective-taking	0.024 (-0.000; 0.136)	0.608 (0.538; 0.688)	0.604 (0.571; 0.636)	0.000	0.000
Elithorne Mazes	0.176 (0.096; 0.284)	0.532 (0.438; 0.636)	0.537 (0.486; 0.604)	0.000	0.000
Mazes	0.134 (0.067; 0.223)	0.577 (0.497; 0.664)	0.537 (0.495; 0.579)	0.000	0.000
Variance captured by the first order factors			**Loading on second order factor**	**A and E variance captured by the general spatial ability factor**	

Navigation latent factor	0.051 (0.011; 0.122)	0.085 (0.041; 0.145)	0.929 (0.904; 0.954)	0.726	0.138
Object-based latent factor	0.265 (0.198; 0.339)	0.075 (0.028; 0.145)	0.812 (0.776; 0.848)	0.551	0.104
Visualization latent factor	0.000 (0.000; 0.000)	0.000 (0.000; 0.000)	1.00 (1.00; 1.000)	0.837	0.163
**Variance common to all tests captured by the second order Spatial Ability factor**					
**Common factor of Spatial ability**	0.837 (0.779; 0.894)	0.163 (0.110; 0.225)	-	-	-
Model fit indices: AIC = 82828.772; χ_2 = 1681.128 (1040), p = 0.0000; CFI = 0.941; TLI = 0.944; RMSEA = 0.026 ; SRMR = 0.056					

Note: CFI = comparative fit index; TLI = Tucker-Lewis index; RMSEA = root mean square error of approximation, SRMR = standardized root mean square residual; AIC = Akaike Information criterion.

Supplementary Table 8. Associations between the time lag of administration between the two batteries and spatial ability measures.

Measure	***r***	***p***	***N***
Navigation directions	0.015	0.524	1,752
Navigation landmark	0.024	0.321	1,733
Map reading	0.004	0.875	1,702
Route memorizing	-0.003	0.914	1,681
Perspective taking (large scale)	0.050	0.036	0.036
Scanning (large scale)	0.010	0.689	1,696
Cross-section	0.026	0.274	1,828
2D drawing	0.065	0.006	1,831
Pattern assembly	0.063	0.008	1,761
Shapes rotation	0.009	0.733	1,621
Mechanical reasoning	0.025	0.283	1,800
Paper folding	0.039	0.103	1,746
3D drawing	0.044	0.074	1,665
Perspective taking	0.036	0.142	1,711
Elithorn maze	0.035	0.152	1,683
Mazes	0.008	0.730	1,692

Supplementary Figures

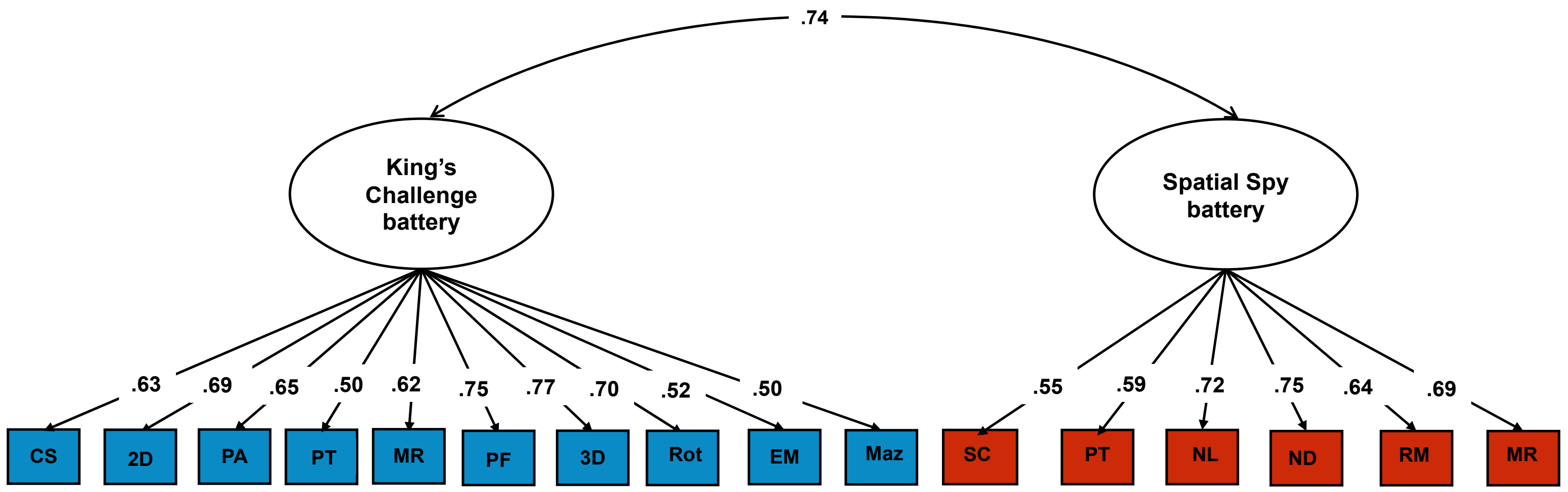

Supplementary Figure 1. Two-factor model of spatial ability separating across the two spatial batteries administered at two different time points and following two different formats (online traditional psychometric assessment – The King's Challenge battery – vs. virtual environment – The Spatial Spy battery). ND = navigation based on directions, NL = navigation based on landmarks, MR = map reading, RM = route memory, PT = perspective taking, SC = scanning, CS = cross-section, 2d = 2d drawing, PA = pattern assembly, EM =Elithorn Mazes, MR = Mechanical Reasoning, PF = paper folding, 3d = 3d drawing, Rot = mental rotation, PT = perspective taking, Maz = mazes; Model fit indices are reported in Table S6.

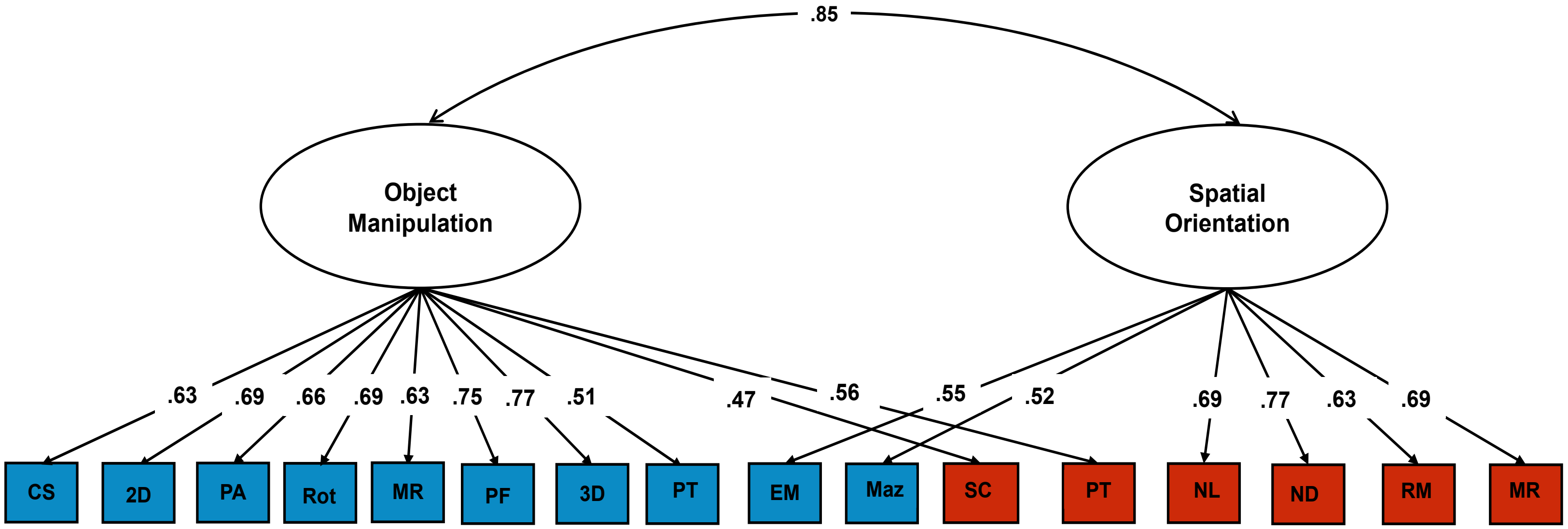

Supplementary Figure 2. Two-factor model of spatial ability separating objects-based and orienting tests combining putatively separate categories of tests administered across the two batteries. ND = navigation based on directions, NL = navigation based on landmarks, MR = map reading, RM = route memory, PT = perspective taking, SC = scanning, CS = cross-sections, 2d = 2d drawing, PA = pattern assembly, EM =Elithorn Mazes, MR = Mechanical Reasoning, PF = paper folding, 3d = 3d drawing, Rot = mental rotation, PT = perspective taking, Maz = mazes; Model fit indices are reported in Table S5.

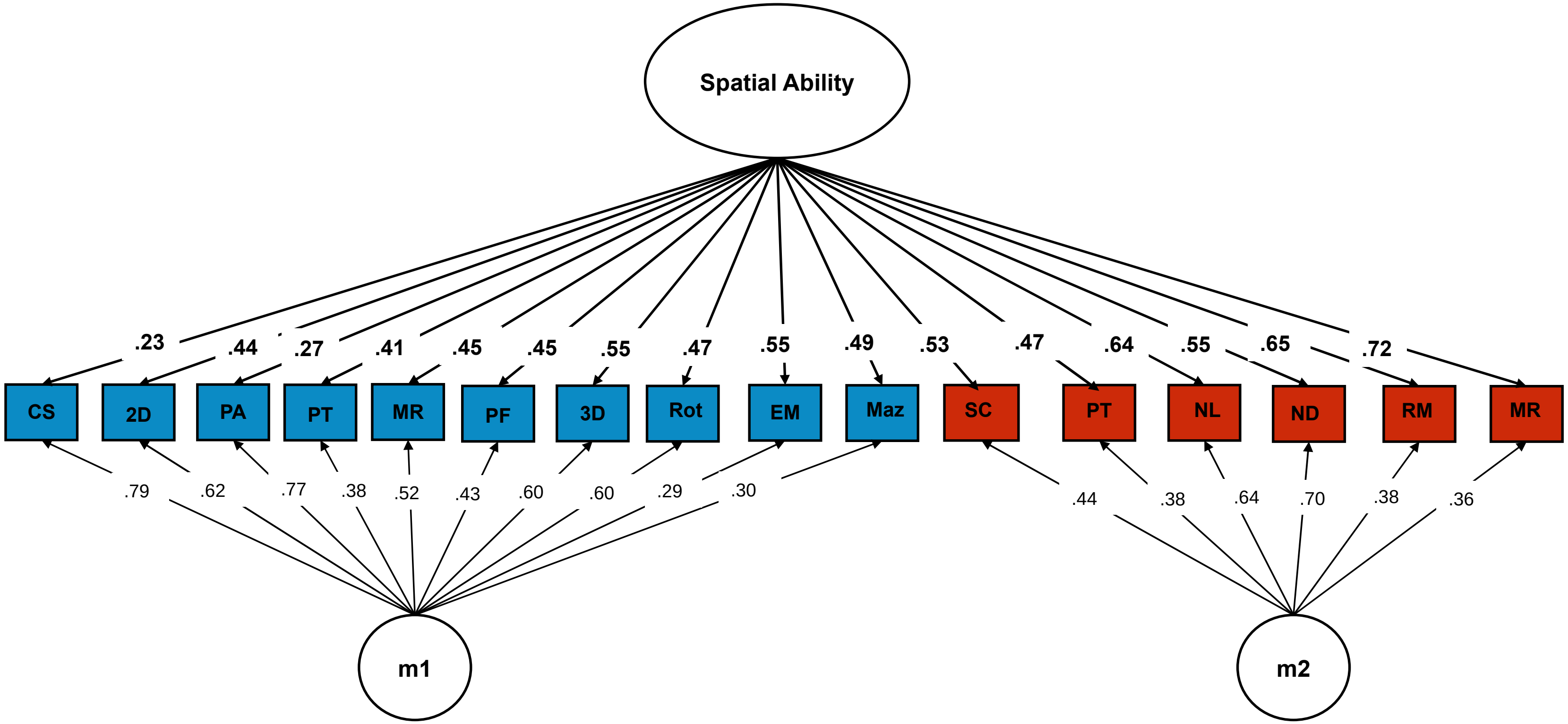

Supplementary Figure 3. Bifactor model. This model allows for the variance common to all tests to load onto a single factor of spatial ability, while separating the variance specific to each of the two batteries (m1 = online traditional psychometric assessment – The King's Challenge battery – vs. m2 = virtual environment – The Spatial Spy battery). The battery-specific variance might reflect a combination of specific ability as well as methodological differences between the two batteries. For this reason, this bifactor model might also be described as a Multi-Trait Multi-Method model. ND = navigation based on directions, NL = navigation based on landmarks, MR = map reading, RM = route memory, PT = perspective taking, SC = scanning, CS = cross-section, 2d = 2d drawing, PA = pattern assembly, EM =Elithorn Mazes, MR = Mechanical Reasoning, PF = paper folding, 3d = 3d drawing, Rot = mental rotation, PT = perspective taking, Maz = mazes; Model fit indices are reported in Table S6.

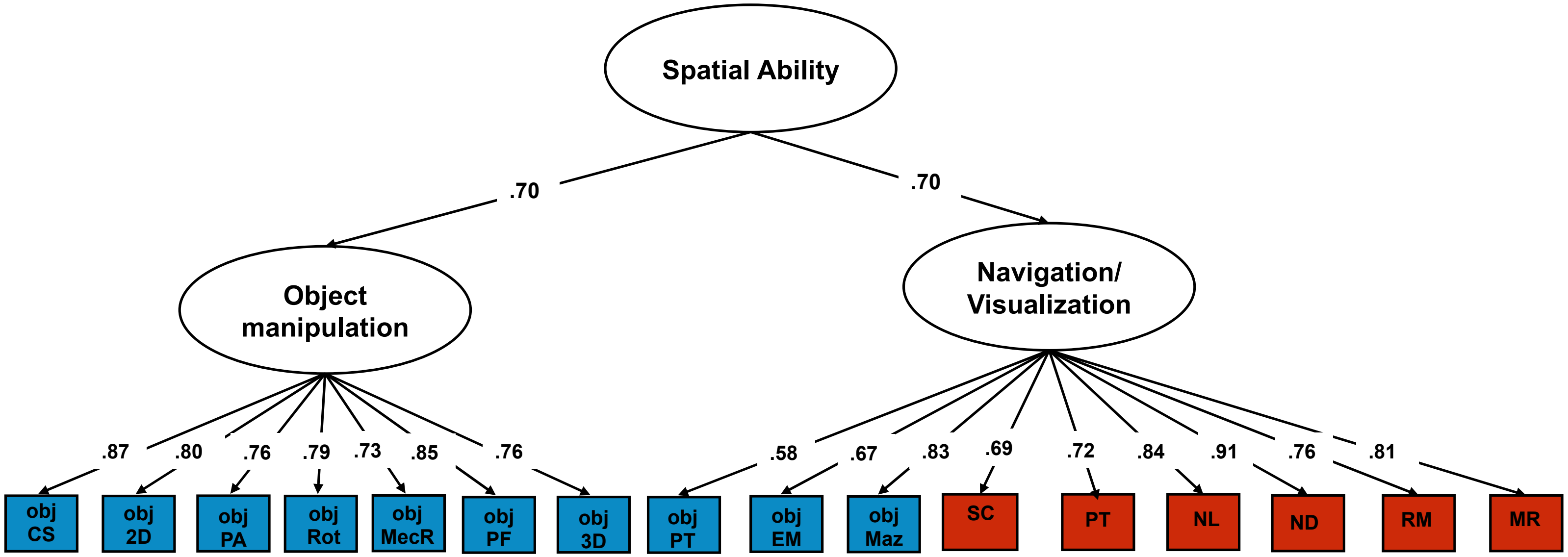

Supplementary Figure 4. Hierarchical model of spatial ability including two first-order factors (object manipulation and navigation/visualization) loading onto a second order general spatial ability factor. ND = navigation based on directions, NL = navigation based on landmarks, MR = map reading, RM = route memory, PT = perspective taking, SC = scanning, CS = cross-section, 2d = 2d drawing, PA = pattern assembly, EM =Elithorn Mazes, MR = Mechanical Reasoning, PF = paper folding, 3d = 3d drawing, Rot = mental rotation, PT = perspective taking, Maz = mazes; Model fit indices are reported in Table S6.

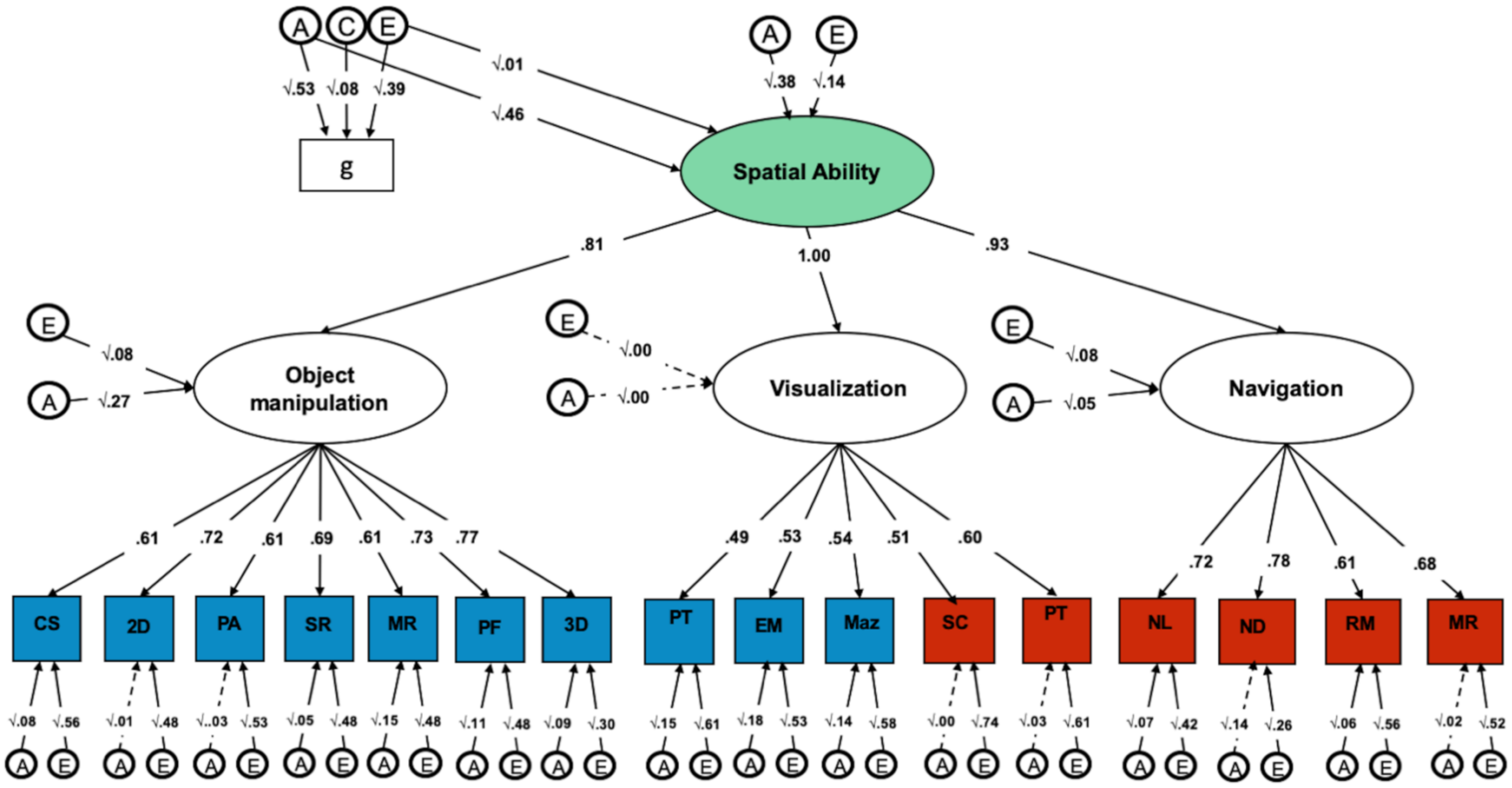

Supplementary Figure 5. Hierarchical common pathway model exploring the genetic and environmental association between *g* and the common spatial ability factor. ND = navigation based on directions, NL = navigation based on landmarks, MR = map reading, RM = route memory, PT = perspective taking, SC = scanning, CS = cross-sections, 2d = 2d drawing, PA = pattern assembly, EM =Elithorn Mazes, MR = Mechanical Reasoning, PF = paper folding, 3d = 3d drawing, Rot = mental rotation, PT = perspective taking, Maz = mazes, *g* = general cognitive ability; Model fit indices are reported in Table S6.

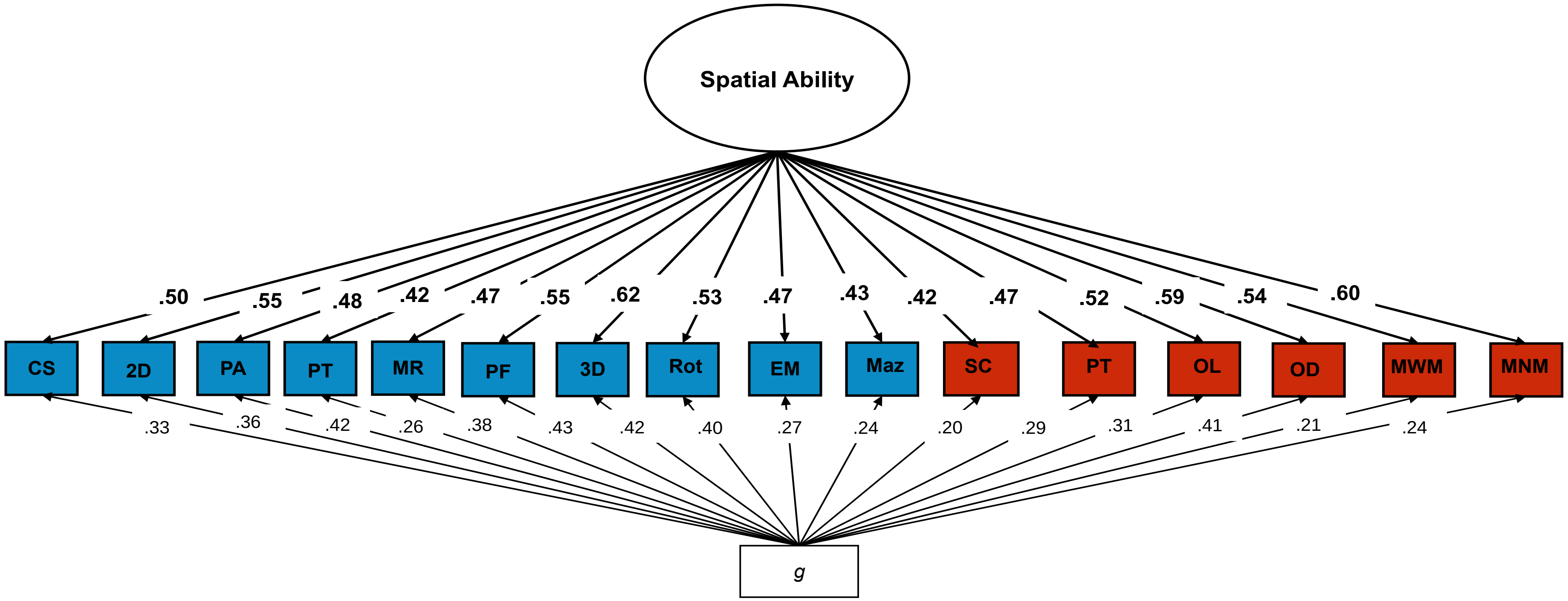

Supplementary Figure 6. One-factor model of spatial ability including the 16 spatial tests accounting for *g* at the level of the indicator (each test); see Table S5 for model fit indices. ND = navigation based on directions, NL = navigation based on landmarks, MR = map reading, RM = route memory, PT = perspective taking, SC = scanning, CS = cross-sections, 2d = 2d drawing, PA = pattern assembly, EM = Elithorn Mazes, MR = Mechanical Reasoning, PF = paper folding, 3d = 3d drawing, Rot = mental rotation, PT = perspective taking, Maz = mazes, *g* = general cognitive ability.

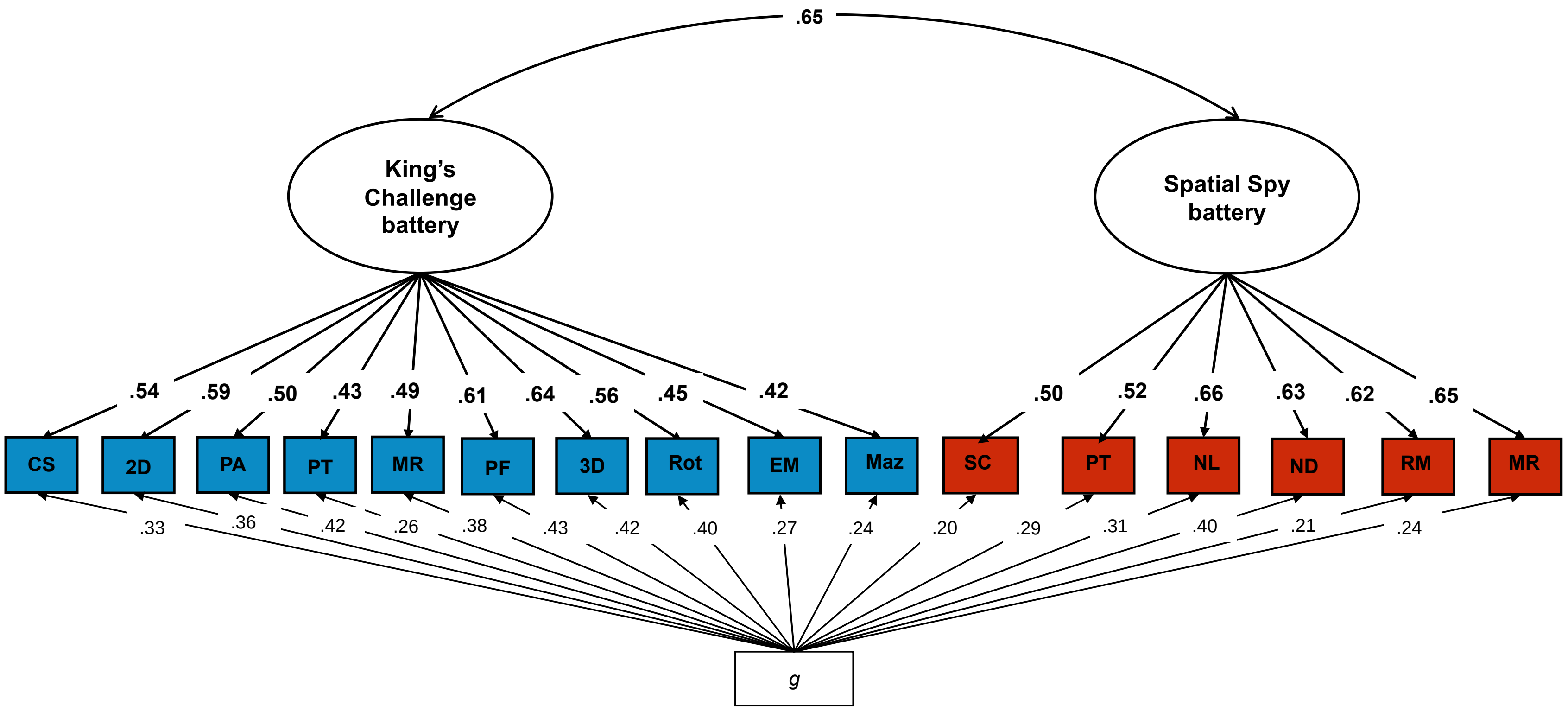

Supplementary Figure 7. Two-factor model of spatial ability separating the two batteries accounting for *g* at the level of the indicators (each test). Model fit indices are reported in Table S5. ND = navigation based on directions, NL = navigation based on landmarks, MR = map reading, RM = route memory, PT = perspective taking, SC = scanning, CS = cross-section, 2d = 2d drawing, PA = pattern assembly, EM =Elithorn Mazes, MR = Mechanical Reasoning, PF = paper folding, 3d = 3d drawing, Rot = mental rotation, PT = perspective taking, Maz = mazes, *g* = general cognitive ability.

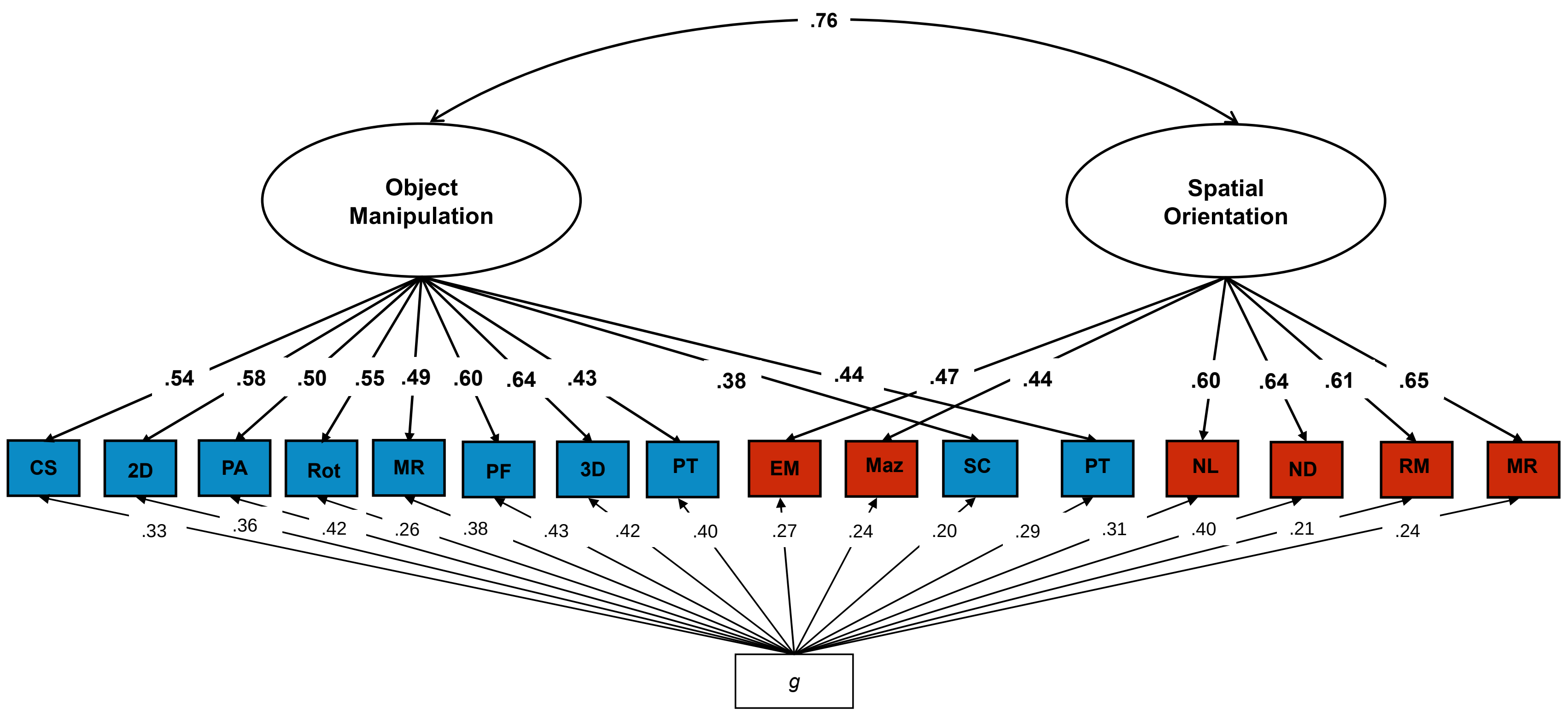

Supplementary Figure 8. Two-factor model of spatial ability separating objects-based and orienting tests combining putatively different aspects of spatial skills across the two batteries accounting for *g* at the level of the indicators (each test). Model fit indices are reported in Table S5. ND = navigation based on directions, NL = navigation based on landmarks, MR = map reading, RM = route memory, PT = perspective taking, SC = scanning, CS = cross-sections, 2d = 2d drawing, PA = pattern assembly, EM =Elithorn Mazes, MR = Mechanical Reasoning, PF = paper folding, 3d = 3d drawing, Rot = mental rotation, PT = perspective taking, Maz = mazes, *g* = general cognitive ability.

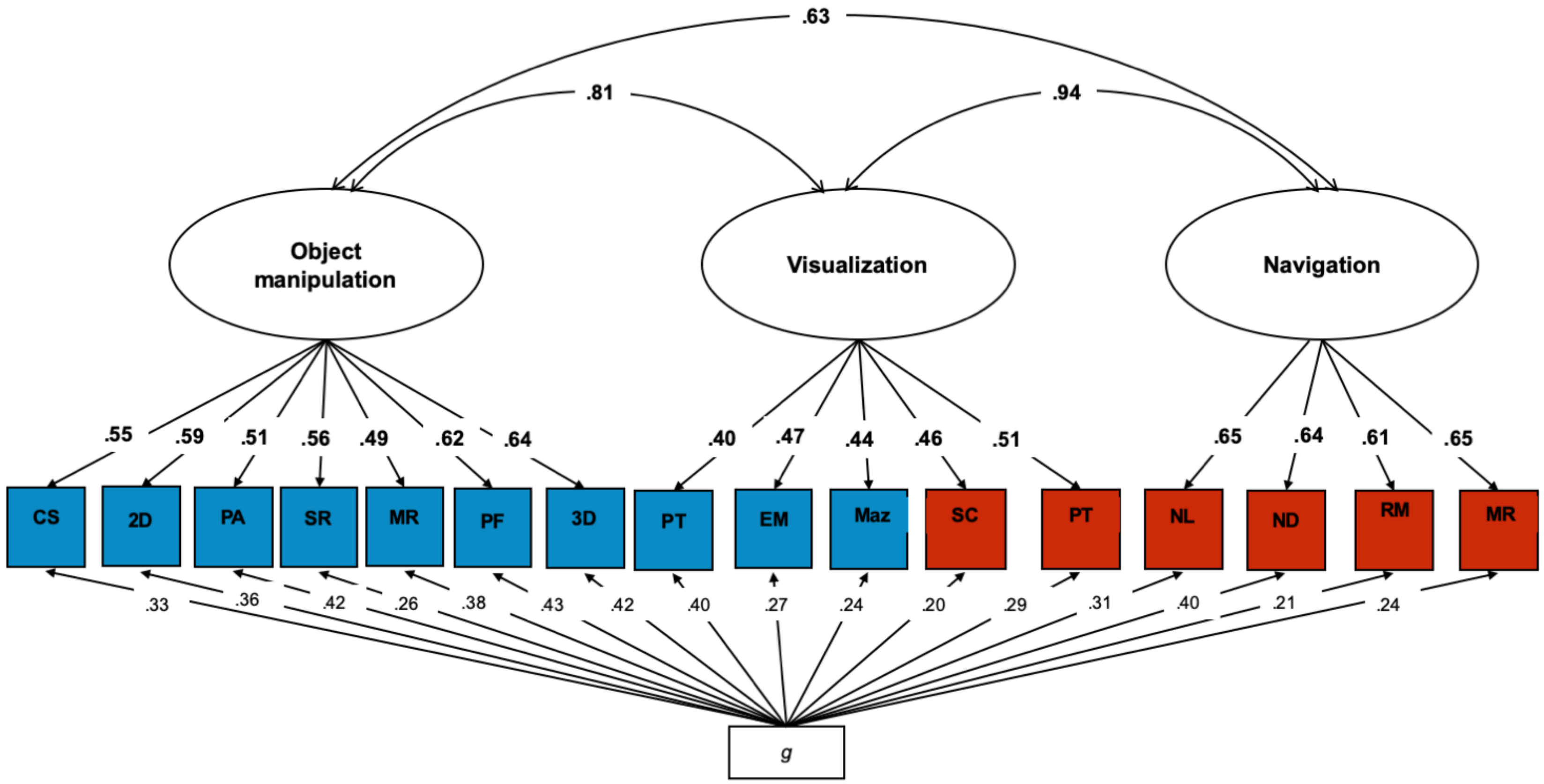

Supplementary Figure 9. Three-factor model of spatial ability separating objects-based, navigation and visualization tests across the two batteries accounting for *g*. Model fit indices are reported in Table S5. ND = navigation based on directions, NL = navigation based on landmarks, MR = map reading, RM = route memory, PT = perspective taking, SC = scanning, CS = cross-sections, 2d = 2d drawing, PA = pattern assembly, EM =Elithorn Mazes, MR = Mechanical Reasoning, PF = paper folding, 3d = 3d drawing, Rot = mental rotation, PT = perspective taking, Maz = mazes, *g* = general cognitive ability.

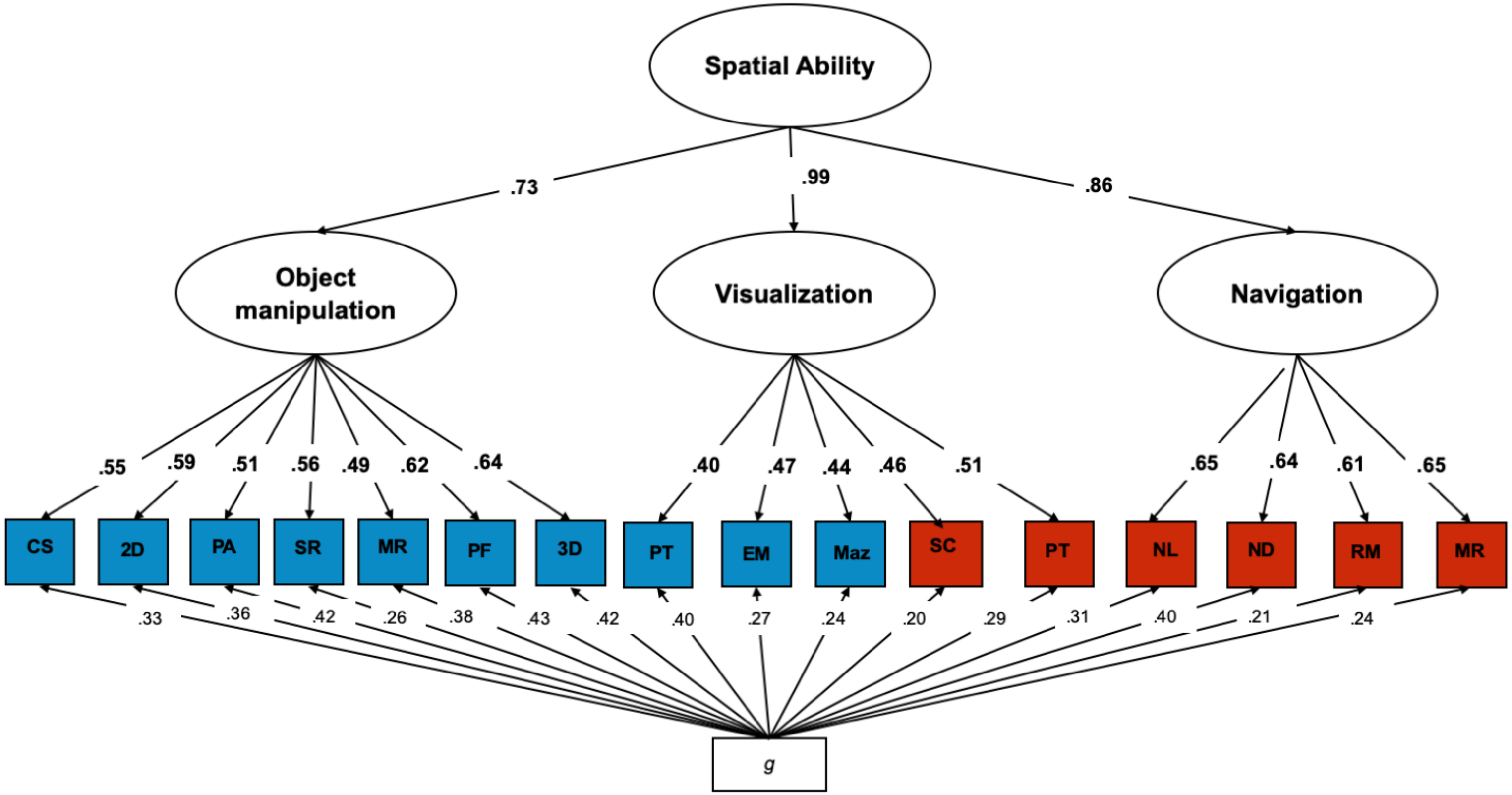

Supplementary Figure 10. Hierarchical model including three first-order spatial factors (Navigation, Object-based and Visualization) and a second-order common factor of Spatial ability, accounting for *g* at the level of the indicators (each test). Model fit indices are reported in Table S5. ND = navigation based on directions, NL = navigation based on landmarks, MR = map reading, RM = route memory, PT = perspective taking, SC = scanning, CS = cross-sections, 2d = 2d drawing, PA = pattern assembly, EM =Elithorn Mazes, MR = Mechanical Reasoning, PF = paper folding, 3d = 3d drawing, Rot = mental rotation, PT = perspective taking, Maz = mazes, *g* = general cognitive ability.

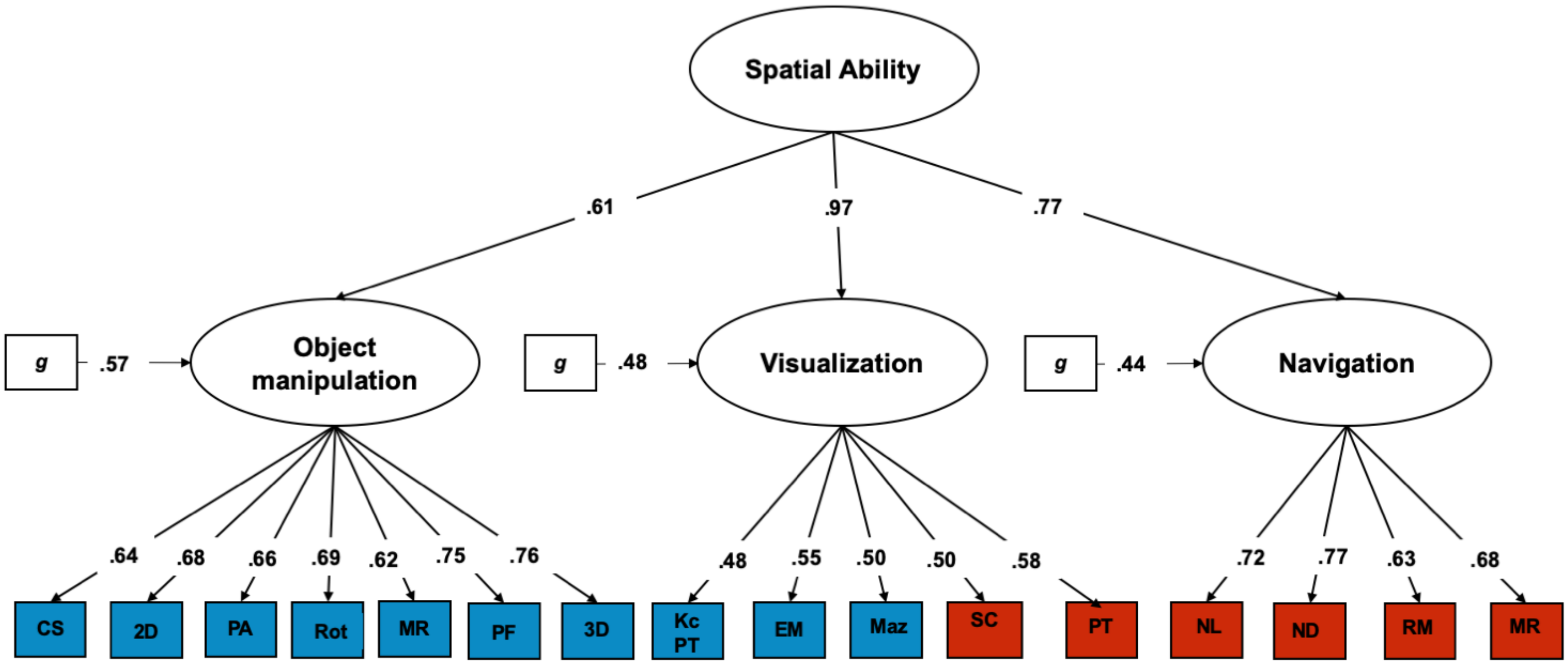

Supplementary Figure 11. Hierarchical model including three first-order spatial factors (Navigation, Object-based and Visualization) and a second-order common factor of Spatial ability, accounting for *g* at the level of the first-order factors. Model fit indices are reported in Table S5. ND = navigation based on directions, NL = navigation based on landmarks, MR = map reading, RM = route memory, PT = perspective taking, SC = scanning, CS = cross-sections, 2d = 2d drawing, PA = pattern assembly, EM =Elithorn Mazes, MR = Mechanical Reasoning, PF = paper folding, 3d = 3d drawing, Rot = mental rotation, PT = perspective taking, Maz = mazes, *g* = general cognitive ability.

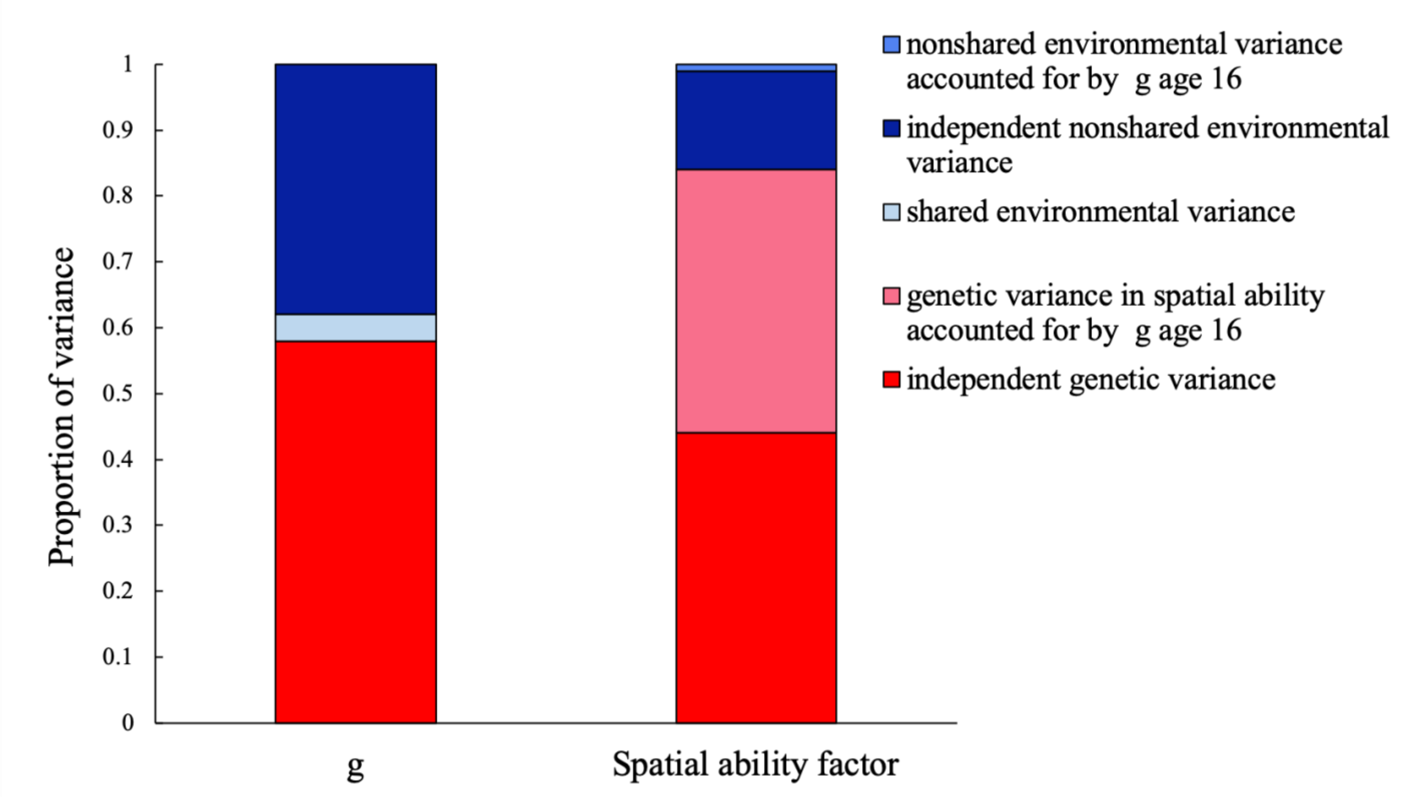

Supplementary Figure 12. Genetic and environmental variance in a measure of g at age 16 and in the common spatial ability factor. For the common spatial ability factor the bar is divided into the genetic and environmental contributions independent of g at age 16 and those that are accounted for by the genetic and environmental variance in g at age 16. Results are from a Cholesky decomposition. The measure of gat age 16 was calculated taking the mean of one verbal (Mill Hill Vocabulary) and one nonverbal (Raven's Progressive Matrices) test.

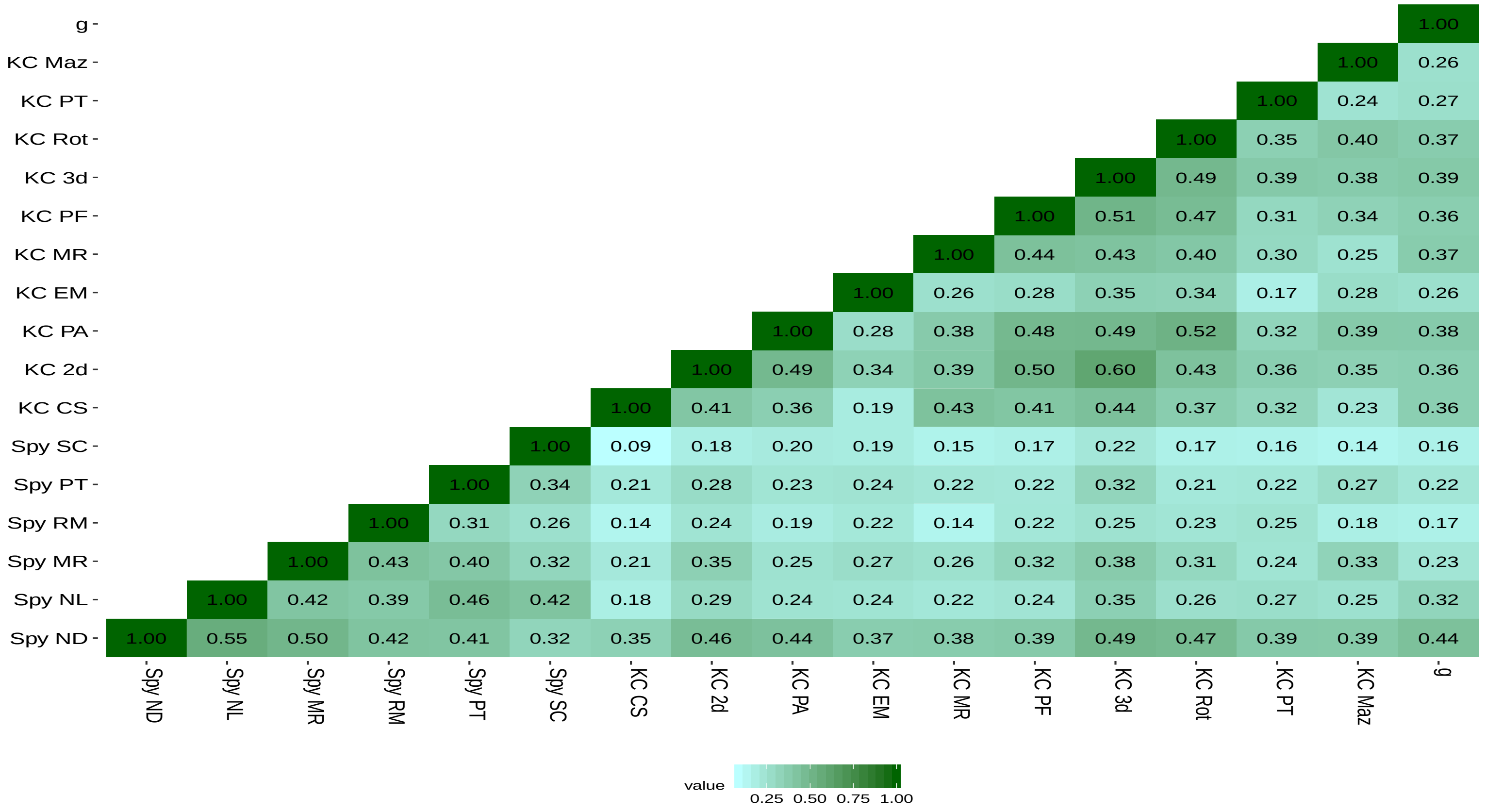

Supplementary Figure 13. Correlations between all spatial tests and general cognitive ability using data from the other half of the phenotypic sample. Spy = Spatial Spy battery (large-scale), KC = King's Challenge battery (small-scale), ND = navigation based on directions, NL = navigation based on landmarks, MR = map reading, RM = route memory, PT = perspective taking, SC = scanning, CS = cross-sections, 2d = 2d drawing, PA = pattern assembly, EM =Elithorn Mazes, MR = Mechanical Reasoning, PF = paper folding, 3d = 3d drawing, Rot = mental rotation, PT = perspective taking, Maz = mazes, *g* = general cognitive ability. All correlations were significant at the $p < .001$ level; variables were residualized for age and sex and standardized prior to analysis.

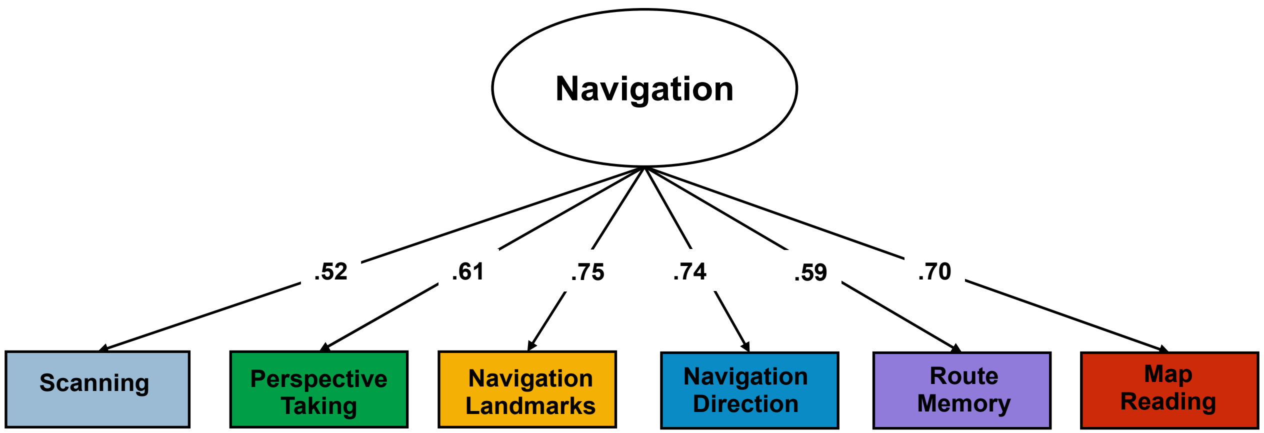

Supplementary Figure 14. Factor structure of navigation abilities conducted examining the other half of the sample; CFI = 0.968, TLI = 0.947, RMSEA = 0.073, SRMR = 0.027.

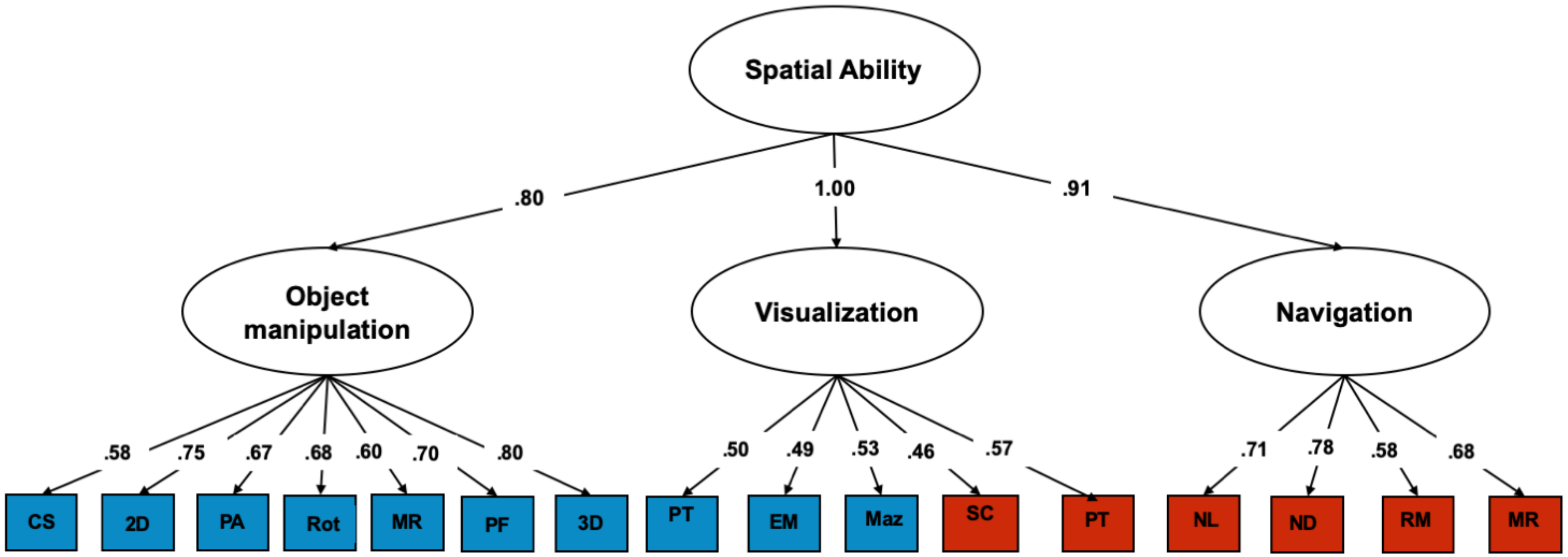

Figure S15. Hierarchical model of spatial abilities conducted in the other half of the sample; CFI = 0.939, TLI = 0.928, RMSEA = 0.046, SRMR = 0.050.
